# The Dutch Body Shape Questionnaire among patients with binge-eating disorder: psychometrics and norms of the full version (BSQ34) and the short version (BSQ8C)

**DOI:** 10.1007/s40519-024-01699-9

**Published:** 2024-11-19

**Authors:** Bernou Melisse, Liselotte de Mooij, Margo de Jonge, Daniela Schlochtermeier, Edwin de Beurs

**Affiliations:** 1 American Center for Psychiatry and Neurology, Al-Manhal, Abu Dhabi, United Arab Emirates; 2 Co-Eur, P.O. box 30514, 3503AH Utrecht, The Netherlands; 3 Department of Clinical Psychology, Utrecht University, PO Box 80140, 3508 TC Utrecht, The Netherlands; 4 Department of Medical and Clinical Psychology, Tilburg University, Postbus 90153, 5000 LE Tilburg, The Netherlands; 5 Novarum Center for Eating Disorders & Obesity, Laan van de Helende Meesters 2, 1186 AM Amstelveen, The Netherlands; 6 Section Clinical Psychology, Leiden University, Wassenaarseweg 52, 2333 AK Leiden, The Netherlands; 7 Research Department, Arkin Mental Health Institute, Klaprozenweg 111, 1033 NN Amsterdam, The Netherlands

**Keywords:** Body Shape Questionnaire, Binge-eating disorder, Body-shape dissatisfaction, Psychometric properties, Normative data

## Abstract

**Purpose:**

This study examined the psychometric properties and provided normative data of the Dutch Body Shape Questionnaire (BSQ34) and its shortened BSQ8C among patients with binge-eating disorder.

**Methods:**

The two versions of the BSQ were administered to patients with binge-eating disorder (*N* = 155) enrolled for treatment, and to a community sample (*N* = 333). The translation and back-translation of the BSQ were performed by translators with and without eating-disorder expertise. Internal consistency, concurrent validity, test–retest reliability, incremental validity, and sensitivity to change were determined. A receiver-operating-characteristic curve-analysis was used to establish criterion-related validity, for which the Eating Disorder Examination—Shape concern subscale, was used. Uni-dimensionality of the instrument was investigated with confirmatory factor analysis. Norms (population-based *T*-scores and clinical percentile-scores) were determined.

**Results:**

The psychometric properties of the BSQs were satisfactory. The BSQ34 discriminated well in body-shape dissatisfaction between patients with binge-eating disorder and the community sample (area-under-the-curve value = 0.91–0.98) and had a unidimensional factor structure. Comparing structural invariance between both samples revealed that scaler invariance was not supported, indicating that items may be interpreted differently by patients with binge-eating disorder and subjects from the community. Analyses were repeated for the BSQ8C, which yielded similar results.

**Conclusion:**

The results indicated that both versions of the BSQ appeared suitable to screen for body-shape dissatisfaction among patients with binge-eating disorder. The BSQ34 supplies valuable information on the various types of concerns respondents have, which are critical to consider in clinical settings; the BSQ8C is recommended as a short screening tool.

*Level of evidence*: Level III: Evidence obtained from well-designed cohort or case–control analytic studies.

**Supplementary Information:**

The online version contains supplementary material available at 10.1007/s40519-024-01699-9.

## Introduction

Body-shape dissatisfaction, defined as “a subjective negative evaluation of one’s physical body”, is a core mechanism of eating disorders [1]. Patients with higher levels of body-shape dissatisfaction benefit less from treatment than patients with lower levels of body-shape dissatisfaction [2–4]. Therefore, one of the aims of eating disorder treatment is to reduce body-shape dissatisfaction [5]. The eating disorder section of the DSM-5 includes anorexia nervosa, bulimia nervosa, and binge-eating disorder (BED), which is most recently included. According to the DSM-5, overevaluation of shape and weight and body-shape dissatisfaction are not necessarily features of BED [6, 7]. BED is characterized by recurrent episodes of binge eating, and the binge-eating episodes are accompanied by a sense of lack of control and feelings of guilt, shame, and disgust. The binge-eating episodes are not followed by inadequate compensatory behavior, such as self-induced vomiting and laxative-misuse [6, 8]. Therefore, most patients with BED have excess-weight [9]. With an estimated life-time prevalence of 2–5%, BED is the most common eating disorder [10, 11]. Moreover, it is estimated that around 30% of the general population with excess-weight who seek weight loss treatment, might meet the diagnostic criteria for BED [11, 12].

Studies show that individuals with excess-weight have higher levels of body-shape dissatisfaction and eating disorder pathology [13, 14]. In addition, recent studies found that 82% of the patients with BED have clinical levels of body-shape dissatisfaction [15, 16], and several studies suggest that body-shape dissatisfaction is comparable between patients with BED and other eating disorders, such as anorexia and bulimia nervosa [17, 18]. Moreover, body dissatisfaction is associated with psychological distress, manifesting in a range of comorbid conditions, including anxiety and depression [19–24]. These issues can severely impair individuals’ well-being and diminish their quality of life. Some researchers have suggested to include overevaluation of shape/weight and body-shape dissatisfaction as a criterion for BED in future revisions of the DSM-5 [17, 25, 26].

The ability to screen for body-shape dissatisfaction could help to assess whether this is present in a BED patient. Subsequently, clinicians can decide whether body-shape dissatisfaction is an appropriate target for intervention within a broader BED treatment [5]. In the Netherlands, the shape concern subscale of the Eating Disorder Examination (EDE-SC) [27, 28] is currently the only assessment tool available to assess body-shape dissatisfaction. However, the Eating Disorder Examination interview (EDE) has several disadvantages, as only trained interviewers can administer it, and its administration is relatively time consuming. A self-report questionnaire for the measurement of body-shape dissatisfaction would help overcome these disadvantages and offer a much-needed alternative.

In 1987, Cooper et al., developed the BSQ-34 to assess concerns about body-shape reported by patients with eating disorders. The Body Shape Questionnaire (BSQ) [29] is used to measure body-shape dissatisfaction [30]. It has 34 items (BSQ34), but various short versions (8A, 8B, 8C, 8D, 14, 16A, 16B) measuring the same construct, have been developed and evaluated [31]. Of these, the BSQ8C shows high sensitivity to change during therapy and this version appears superior over the other shortened versions [32]. Both the BSQ34 and BSQ8C are adapted for use in various countries [30–35]. They have a unidimensional factor structure, and strong psychometric properties, such as high test–retest reliability, internal validity, and convergent validity.

The ability to measure body-shape dissatisfaction could help clinicians to decide whether body-shape dissatisfaction should be addressed during treatment of patients with BED [5]. This requires appropriate cut-off values for screening purposes. Furthermore, availability of a valid Dutch shortened BSQ could be used to measure reduction of body-shape dissatisfaction after treatment. Moreover, norms need to be established. Based on the Item Response Theory (IRT) analysis, factor scores could be used to obtain normalized standard scores (*T*-scores) [36], and percentile scores can be established. Both will offer a conversion of raw BSQ scores into common metrics, which will ease interpretation of scores on the BSQ, facilitate communication with patients, and increase the applicability of the BSQ [37].

In sum, the first aim of this study is to evaluate the psychometric properties (internal consistency, concurrent validity, test–retest reliability, incremental and criterion-related validity, sensitivity to change, and uni-dimensionality) of the BSQ34, and the BSQ8C, which have been first translated into Dutch, among patients with BED. Another aim is to determine norms (population-based *T*-scores and clinical percentile scores), as they enable the measurement of changes in body-shape dissatisfaction over time. In addition, it is also important to examine whether the BSQ indeed measures body-shape dissatisfaction among patients with an eating disorder. Therefore, the criterion-related validity of both versions of the BSQ will be assessed by examining whether the BSQ accurately discriminates between individuals with (*N* = 155), or without an eating disorder (*N* = 333). It is hypothesized that all the psychometric properties of the Dutch BSQs are satisfactory, that a unidimensional factor structure is supported, and that the BSQs accurately discriminate between patients with BED and a community sample.

## Method

### Procedure

The BSQ34 was compared to the Eating Disorder Examination-Shape Concern (EDE-SC) subscale to examine if the BSQ accurately measures body-shape dissatisfaction. Data were collected in two convenience samples. Data from patients with BED (sample 1) were pre-treatment data collected as part of a randomized controlled trial examining the efficacy of two web-based versions of Cognitive Behavior Therapy- Enhanced (CBT-E) for BED: a guided self-help version (GSH-CBT-E) or treatment as usual (TAU-CBT-E) [38–40]. Further study details were extensively explained elsewhere [41]. All patients with BED who were referred for specialized treatment were invited to participate in the study. Eligible patients received verbal and written study information during an introductory session, including a description with an explanation of the research goals and information about their participation. After patients had provided informed consent, the EDE [42] was used to assess the severity of BED. Subsequently, the BSQ34, as well as the EDE-SC were completed by the patients at the start, and to measure sensitivity to change at the end of treatment. The EDE and EDE-SC were administered by phone interview, and the BSQ was administered through a web-based application. Recruitment took place between January 2022 and March 2023.

Data were also collected in a community-based convenience sample (sample 2). Participants were recruited through social media (Twitter, Facebook, Instagram) and leaflets presented at general practitioners' offices. Additionally, 20 students of author BM recruited participants through their personal network as part of their bachelor thesis. Finally, students of Utrecht University could receive mandatory study credits in exchange for participation. Recruitment of sample 2 took place between September 2021 and May 2023. To assess test–retest reliability, sample 2 completed the BSQ at the start of the study and again 3 weeks later. The EDE-SC was administered once, concurrent with the first BSQ assessment. All assessments were processed in Castor EDC [43], which is ISO 27001/27002/9001 and NEN 7510 certified. This study’s design and its analysis were not preregistered.

### Participants

Eligible patients in sample 1 were aged 18 or over, had a DSM-5 BED diagnosis [6], and a BMI between 19.5 and 40, since CBT-E was explicitly designed for non-underweight patients with a BMI up to 40 [44]. Sufficient proficiency in Dutch and internet access were required for participation in the trial. An exclusion criterion was an eating disorder other than BED. Participants of sample 2 were literate in Dutch, aged ≥ 18, and had internet access. Participants provided informed consent and completed anonymously online a set of self-report questionnaires including the BSQ34 and provided some demographic information.

### Measures

#### Body Shape Questionnaire

The BSQ34 is a self-report questionnaire measuring body-shape dissatisfaction in the previous 28 days. A total of 34 items are answered on a 6-point Likert frequency scale (1: never, to 6: always) related to the desire to lose weight, fear of weight gain, and self-devaluation related to physical-appearance [29]. The total score is calculated as the sum of all items (range 34–204). The proposed clinical cut-off score for the British original is > 110, indicating clinically significant body-shape dissatisfaction in the context of an eating disorder. The BSQ8C, a shortened ‘alternate’ form, measures the same construct and comprises 8 items: 4, 6, 13, 16, 19, 23, 29, and 33 (range is 8–48) [45], and has a recommended cut-off score of > 26 [29]. Both versions of the BSQ have good psychometric properties, such as good test–retest reliability (*r* = 0.88, and 0.95 for the BSQ34 and the BSQ8C, respectively), and high internal consistency (Cronbach’s *α* = 0.96, and 0.91, respectively) [30, 32, 34].

To establish a validated Dutch version (See Supplementary Materials), the British original was independently translated twice: by author BM and by an official translator without specific eating disorder expertise. The two Dutch translations were examined and compared by a third translator, and then discussed until a consensus version was established. This procedure was repeated by author LdM and two other translators to obtain a back translation. Discrepancies among all translations were discussed and resolved by consensus among the translators [46].

A pilot study among *N* = 40 Utrecht University students was conducted in March 2021. They rated comprehension of all items on a 4-point scale (1: I do not understand this at all, 4: completely clear). Participant feedback in this pilot indicated that the quality of the translation was satisfactory (mean comprehensiveness rating of all items: *M* = 3.60, *SD* = 0.30).

#### Eating disorder examination 17.0

The Dutch version of the Eating Disorder Examination (EDE) was employed in the present study [28]. The EDE is a widely used semi-structured interview assessing severity of eating disorder pathology in the previous 28 days. All items are scored on a 7-point Likert scale (0: feature was absent, to 6: feature was markedly present/present every day) [27, 28]. The EDE exists of four subscales: weight concern, eating concerns, dietary restraint and shape concern. The global score is calculated as the average of its subscales. Jansen et al., (2000) have reported good psychometric properties of their Dutch translation of the EDE. In sample 1, 98% (*n* = 152/155) scored above the cutoff of 1.77.

The shape concern subscale (EDE-SC) consists of eight items (23, 24, 25, 27, 28, 29, 30, 31) measuring shape concerns in the context of an eating disorder. The EDE-SC has a cut-off score of > 2.43 [27, 28]. In sample 1, 93% (*n* = 144/155) scored above the cutoff. Internal consistency of the EDE-SC in sample 1 was acceptable (Cronbach’s *α* = 0.70, McDonalds *w* = 0.62), and an Exploratory Factor Analyses indicated a unidimensional factor structure for the shape concern subscale with item loadings between 0.10-0.50. Internal consistency of the EDE-SC in sample 2 was excellent (Cronbach’s *α* = 0.91, McDonalds *w* = 0.91).

#### Clinical Impairment Assessment (CIA)

The Clinical Impairment Assessment (CIA) measures impaired psychosocial functioning due to eating disorder pathology during the previous 28 days. The CIA is a 16-item self-report questionnaire, rated on a 4-point Likert-scale (0: not at all; 3: a lot). The global score is calculated as the sum of all items (range 0–48), with a cutoff of > 16 [47]. Internal consistency of the CIA was good in present study (Cronbach’s *α* = 0.76, McDonalds ω = 0.78).

#### Comorbid psychopathology and demographics

Various demographic and eating disorder related variables were administered through self-report (age, gender, level of education, professional status, civil status, if they received eating disorder treatment in the past, and the use of psychopharmacology). Information regarding the presence of comorbid psychopathology was provided by self- report. If present, comorbid psychopathology was assessed by a psychiatrist or clinical psychologist with the SCID-5-CV [48]. When patients were diagnosed with comorbid psychopathology elsewhere, their diagnoses were copied from their psycho-diagnostic reports.

### Ethics

The trial (sample 1) was registered at the Dutch Trial Registry (https://trialsearch.who.int/Trial2.aspx?TrialID=NL9555). The trial approval was given in May 2021 (NL 76368.100.21) by the Medical Research Ethics Committees United. Study approval for sample 2 was given in March 2021 (21.0581) by the Faculty Support Office Ethics Committees of Utrecht University. All data were anonymized before analysis, and all the participants provided informed consent.

### Statistical analyses

Item scores were inspected regarding their mean, *SD* and frequency distribution by examination of skewness and kurtosis. Internal consistencies of the BSQs among both samples was measured by Cronbach’s *α* and McDonald’s ω (≥ 0.70 was considered acceptable, ≥ 0.90 excellent) [49–51]. Concurrent validity was examined by bivariate correlations between the BSQs and the EDE-SC [52]. Test–retest reliability was measured by a dependent sample *t*-test. In addition, incremental validity was assessed by examining whether BSQ scores were correlated with the EDE global scores through a regression analyses. Criterion-related validity, comparing BSQ scores among a clinical and a community sample, was examined using an independent sample *t*-test. In addition, the criterion-related validity of the Dutch BSQ34 and BSQ8C was examined by a receiver-operating-characteristic (ROC) analysis. The ROC analyses were first performed among sample 1 and 2 separately, with EDE-SC status as criterion. Subsequently, the ROC analysis was repeated among a combined sample of sample 1 and 2, with sample type (patients with BED versus community population) as the criterion. Differences between samples were examined for each variable with a *χ*^2^-test or with an independent samples *t*-test. Furthermore, sensitivity and specificity of both versions of the BSQ were established regarding the presence of body-shape dissatisfaction as assessed by the EDE-SC. The area under the curve (AUC) was calculated: An AUC ≥ 0.90 meant high, 0.70–0.90 moderate, and 0.50–0.70 low accuracy in predicting EDE-SC status. Sensitivity to change was measured by a dependent sample *t*-test before and after eating disorder treatment. Moreover, among sample 1, a confirmatory factor analysis (CFA) was performed to investigate the uni-dimensionality of the BSQs. Invariance of the unidimensional structure of the BSQ34 and the BSQ8C across sample 1 and sample 2 was investigated with a multi-group CFA measurement [53]. Finally, as described elsewhere in detail, an IRT-based transformation of scores was performed [36, 37] on the data of the community sample to arrive at community-based normalized *T*-scores. First, an IRT model was fitted to the data of samples 1 and 2, and factor scores (thetas with *M* = 0, *SD* = 1 for sample 2) were calculated. Secondly, these standard scores were converted into *T*-scores with *T* = 10*Z + 50. With curve fitting (non-linear least squared) a function was derived to compute *T*-scores from raw scores [54]. Cut-off values between 55 and 60 were proposed for *T*-scores. Consequently, the appropriateness of these cut-off value for clinically significant body-shape dissatisfaction in the context of BED was investigated. The results were reported in line with the Standards for Reporting of Diagnostic Accuracy Studies guidelines [55]. Data were analyzed with SPSS version 29 [56], *R* and *R* packages Lavaan, version 0.6–5 [57], nls and nlstools [53], and mirt [58].

### Data availability statement

Materials are available upon reasonable request.

## Results

### Participants

For sample 1,177 potential participants were recruited, of which *n* = 22 did not meet the inclusion criteria; resulting in a sample of *N* = 155 participants diagnosed with BED, who agreed to participate and signed informed consent. There were no missing data. Most participants were women (*n* = 135/155, 87.1%), and mean age and BMI were 37.5 (*SD* = 11.8) years, and 34.1 (*SD* = 5.0) kg/m^2^, respectively. Only the BSQ8C scores were higher among women compared to men (BSQ34: *t* (154) = 3.4, *p* = 0.060; BSQ8C: *t* (154) = 2.8, *p* = 0.014). A total of *n* = 118/155 (76%) completed treatment within this studies’ timeframe and therefore provided completed measures after treatment. For sample 2, *N* = 333 participants were recruited, *n* = 237/333 (71.2%) were women. Mean age and BMI were 32.7 (*SD* = 16.5) years, and 24.5 (*SD* = 7.8) kg/m^2^. A total of *n* = 186/333 (55.6%) participants completed the second set of measurements 3 weeks after. Table 1 displays the demographics of both samples. Table 1 shows that gender, marital status, highest level of education, eating disorder treatment in the past, comorbid diagnoses, use of psychopharmacology and the scores on the self-report measures differed between the samples.

**Table 1:** Demographics of patients with BED (*N* = 155), a convenience sample (*N* = 333), and the general Dutch population

	Clinical sample	Community sample	*p*	Cohen’s d [CI95]	General Dutch population (CBS, 2023)
	*n* = 155	*n* = 333			
Age, *M (SD)*	37.5 (11.8)	32.7 (16.5)	0.731	0.33 [0.14, 0.53]	42.4
Baseline BMI, *M (SD)*	34.1 (5.0)	24.5 (7.8)	0.568	1.47 [1.25, 1.68]	25.8
Gender, *n* (%)			< 0.001		
Women	135 (87.1%)	237 (71.2%)			(50.3%)
Men	20 (12.9%)	96 (28.8%)			(49.7%)
Highest level of education, *n* (%)			0.010		
No education	1 (0.6%)	0 (0%)			(23.1%)^a^
Primary school	3 (1.9%)	2 (0.6%)			
Lower vocational education	1 (0.6%)	1 (0.3%)			
Lower general secondary education	11 (7.1%)	6 (1.8%)			
Senior general secondary education/ university preparatory education	13 (8.4%)	139 (41.7%)			(30.3%)^a^
Secondary vocational education	38 (24.5%)	25 (7.5%)			
Higher professional education	57 (36.8%)	75 (22.5%)			(28.6%)^1^
University	31 (20.0%)	85 (25.5%)			
Unknown					(18.0%)^1^
Profession, *n* (%)			0.726		
Student	18 (11.6%)	171 (51.4%)			
Employed	110 (71.0%)	133 (39.9%)			(53.5%)
Volunteer job	5 (3.2%)	2 (0.6%)			
Unemployed	8 (5.2%)	6 (1.8%)			(20.1%)
Other	14 (9.0%)	21 (6.3%)			
Marital status, *n* (%)			< 0.001		
Single	78 (50.3%)	223 (70.0%)			(56.2%)
Registered partnership	19 (12.3%)	16 (4.8%)			
Married	45 (29.0%)	77 (23.1%)			(36.9%)^b^
Divorced	13 (8.4%)	7 (2.1%)			(6.9%)
Eating disorder treatment in the past, *n* (%)			< 0.001		NA
Yes	21 (13.5%)	12 (3.6%)			
No	133 (85.8%)	321 (96.4%)			
Unknown	1 (0.6%)				
Comorbid diagnosis, *n* (%)			0.006		NA
No	77 (49.7%)	225 (67.6%)			
I don't know	17 (11.0%)	41 (12.3%)			
Mood disorder	19 (12.3%)	13 (3.9%)			
Anxiety disorder	8 (5.2%)	8 (2.4%)			
Attention deficit (hyperactive) disorder	11 (7.1%)	19 (5.7%)			
Posttraumatic stress disorder	3 (1.9%)	4 (1.2%)			
Personality disorder	6 (3.9%)	1 (0.3%)			
Autism	2 (1.3%)	8 (2.4%)			
Other	10 (6.5%)	12 (3.6%)			
Unknown	1 (0.6%)				
Use of psychopharmacology, *n* (%)			< 0.001		NA
Yes	36 (23.2%)	17 (5.1%)			
No	118 (76.1%)	316 (94.9%)			
Unknown	1 (0.6%)				
Eating disorder pathology (EDE), *M* (SD)					NA
Global score	3.0 (0.9)	NA			
Dietary restraint	2.0 (1.3)	NA			
Eating concern	2.5 (1.3)	NA			
Weight concern	3.5 (1.1)	NA			
Shape concern	3.8 (1.2)	NA			
Eating disorder pathology (EDE-Q global score), *M* (SD)	3.4 (1.0)	1.3 (1.1)	< 0.001	2.00 [1.77, 2.23]	NA
Binge eating (EDE), *M* (SD)					
Objective episodes	4.9 (9.9)	NA			
Subjective episodes	15.6 (22.4)	NA			
Days with objective episodes	4.6 (8.4)	NA			
Days with subjective episodes	9.9 (10.8)	NA			
Secondary pathology (CIA), *M* (SD)					NA
Global score	23.09 (9.4)	5.44 (7.5)	< 0.001	2.08 [1.84, 2.31}	
Body-shape dissatisfaction (BSQ34), *M* (SD)	124.3 (28.5)	67.1 (29.2)	< 0.001	1.98 [1.75, 2.21]	NA
Body-shape dissatisfaction, shortened (BSQ8C), *M* (SD)	32.0 (6.9)	16.2 (7.5)	< 0.001	2.19 [1.96,2.43]	

*M* mean, *SD* standard deviation, *BMI* body mass index, *EDE-Q* Eating Disorder Examination- Questionnaire, *EDE* Eating Disorder Examination, *CIA* Clinical Impairment Assessment, *BSQ* Body Shape Questionnaire

CBS. (2023). *Dutch Central Bureau of Statistics*. Retrieved 05–07 from https://www.cbs.nl/nl-nl/visualisaties/dashboard-bevolking

^a^Lower education includes no education, primary school, lower vocational and lower general secondary education, secondary education includes senior general secondary, university preparatory and secondary vocational education, and higher education includes higher professional education and university

^b^Includes widowed

### Psychometric properties

#### Internal consistency

Table 2 shows that internal consistencies were high (BSQ34: Cronbach’s *α* = 0.95, McDonalds *w* = 0.95; BSQ8C: Cronbach’s *α* = 0.80, McDonalds *w* = 0.80) in sample 1, as well as in sample 2 (BSQ34: Cronbach’s *α* = 0.97, McDonalds *w* = 0.97; BSQ8C: Cronbach’s *α* = 0.91, McDonalds *w* = 0.92).

**Table 2:** Summary of reliability and validity measures of the Dutch BSQ34 and BSQ8C

Sample, *n* participants	Measure	Mean	*SD*	*α*	*ω*	*R* (EDE-SC)	*AUC*	*n,* % above original cutoff †	*n,*% above estimated cutoff ‡
Sample 1^a^	BSQ34	124.3	28.5	0.95	0.95	0.73*	0.90	109, 70.3%	130, 83.9%
(*N* = 155)	BSQ8C	32.0	6.9	0.80	0.80	0.72*	0.91	129, 83.2%	153, 98.7%
Sample 1^a^	BSQ34	124.3	28.5	0.95	0.95	0.73*	0.90	109, 70.3%	130, 83.9%
(*N* = 155)	BSQ8C	32.0	6.9	0.80	0.80	0.72*	0.91	129, 83.2%	153, 98.7%
Sample 2^b^	BSQ34	67.1	29.2	0.97	0.97	0.89*	0.98	37, 11.1%	49, 14.7%
(*N* = 333)	BSQ8C	16.2	7.5	0.91	0.92	0.87*	0.98	45, 13.5%	130, 39.0%
Combined sample^c^	BSQ34	85.2	39.3	0.97	0.96	0.88*	0.91	146, 29.9%	179, 36.7%
(*N* = 488)	BSQ8C	21.2	10.3	0.87	0.88	0.86*	0.93	174, 35.6%	184, 37.7%

*α* Cronbach’s alpha [49], *ω* Omega (hierarchical) (MacDonald, 1999), *BSQ* Body Shape Questionnaire, *SD* standard deviation, *EDE-SC* Eating Disorder Examination- Shape Concern subscale, *AUC* area under the curve

^a^Sample 1: patients with BED’ EDE-SC scores used as criterion

^b^sample 2: community sample EDE-SC scores used as criterion

^c^combined sample: sample 1 and 2, sample type (patients with BED versus community population) used as criterion

^*^ p < .001

^†^Original cut-off: BSQ34 < 110; BSQ8C < 26 [27]

^‡^ Estimated cut-off: BSQ34 < 97; BSQ8C < 16.5

#### Concurrent validity and test–retest reliability

The concurrent validity was supported: BSQ and EDE-SC scores were significantly correlated (for BSQ34: *r* = 0.70, *p* < 0.001; for BSQ8C: *r* = 0.71, *p* < 0.001) both in sample 1, and 2 (for BSQ34: *r* = 0.89, *p* < 0.001; for BSQ8C: *r* = 0.87, *p* < 0.001). A high 3-week test–retest reliability was found (BSQ34 *r* = 0.92, BSQ8C *r* = 0.90) in sample 2.

#### Incremental validity

The BSQ total scores were correlated with the EDE global score in sample 1: (BSQ34: adjusted *R*^2^ = 0.50, *R* = 0.68, *p* ≤ 0.001, *F*(1,154) = 128.5; BSQ8C: adjusted *R*^2^ = 0.44, *R* = 0.66, *p* ≤ 0.001, *F*(1,154) = 119.9). This means that the BSQ can improve the explanation of body-shape dissatisfaction among patients with BED on top of the EDE, compared to solely administering the EDE.

#### Criterion-related validity and sensitivity to change

A high AUC was revealed in sample 1 (BSQ34: *AUC* = 0.90 [0.83–0.97], *p* < 0.001; BSQ8C: *AUC* = 0.91 [0.84–0.98], *p* < 0.001), which indicated that both Dutch BSQs accurately measure body-shape dissatisfaction according to the EDE-SC among patients with BED. Table 2 shows that the results were similar in a sample 2. Table 2 also shows that the BSQs also accurately discriminated between sample 1 and 2 (patients with BED versus community population) when sample type was used as criterion.

The independent sample *t*-test showed that the BSQ scores differed between sample 1 and 2: BSQ34: *t* (486) = 20.3, *p* ≤ 0.001, BSQ8C: *t* (486) = 22.6, *p* ≤ 0.001. In sample 1 sensitivity to change was accurate among both versions (paired samples *t*-test: BSQ34: *t* (117) = 12.3, *p* ≤ 0.001, BSQ8C: *t* (117) = 12.8, *p* ≤ 0.001). Sensitivity to change on the BSQs was in concordance with the sensitivity to change on the EDE-SC subscale (paired samples *t*-test: EDE-SC: *t* (117) = 12.9, *p* ≤ 0.001.

#### Confirmatory factor analysis

The IRT analysis performed on sample 1 showed that a unidimensional model fitted well (χ^2^(527) = 2120.26; RMSEA = 0.079; [0.075–0.082]; SRMSR = 0.041, TLI = 0.982, CFI = 0.908) for the BSQ34. Similar fit indexes were found for the BSQ8C: (χ^2^(20) = 83.80; RMSEA = 0.081; [0.063–0.099]; SRMSR = 0.029, TLI = 0.984, CFI = 0.989). A multi-group CFA showed that, with regard to configural and metric invariance, both BSQs had a sufficiently similar factor structure in sample 1 (patients with BED) and sample 2 (community sample). However, regarding scalar invariance, a significant difference was found [53]. These results indicate that support for the unidimensional factor structures of the BSQ34 and BSQ8C with sufficiently similar loadings of items was found, irrespective of clinical status. Item intercepts were dissimilar, and items may be interpreted differently by both groups.

#### Norms

**Fig. 1:**
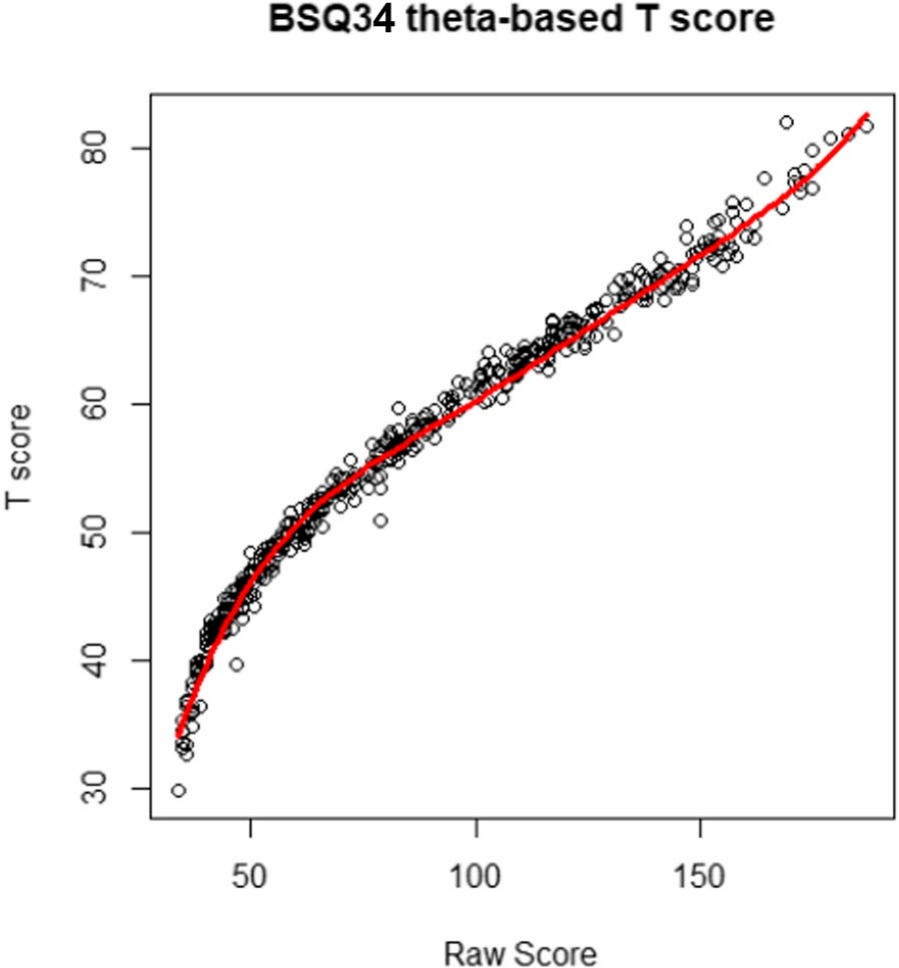
Relationship between raw scores and theta-based T-scores on the BSQ34

**Fig. 2:**
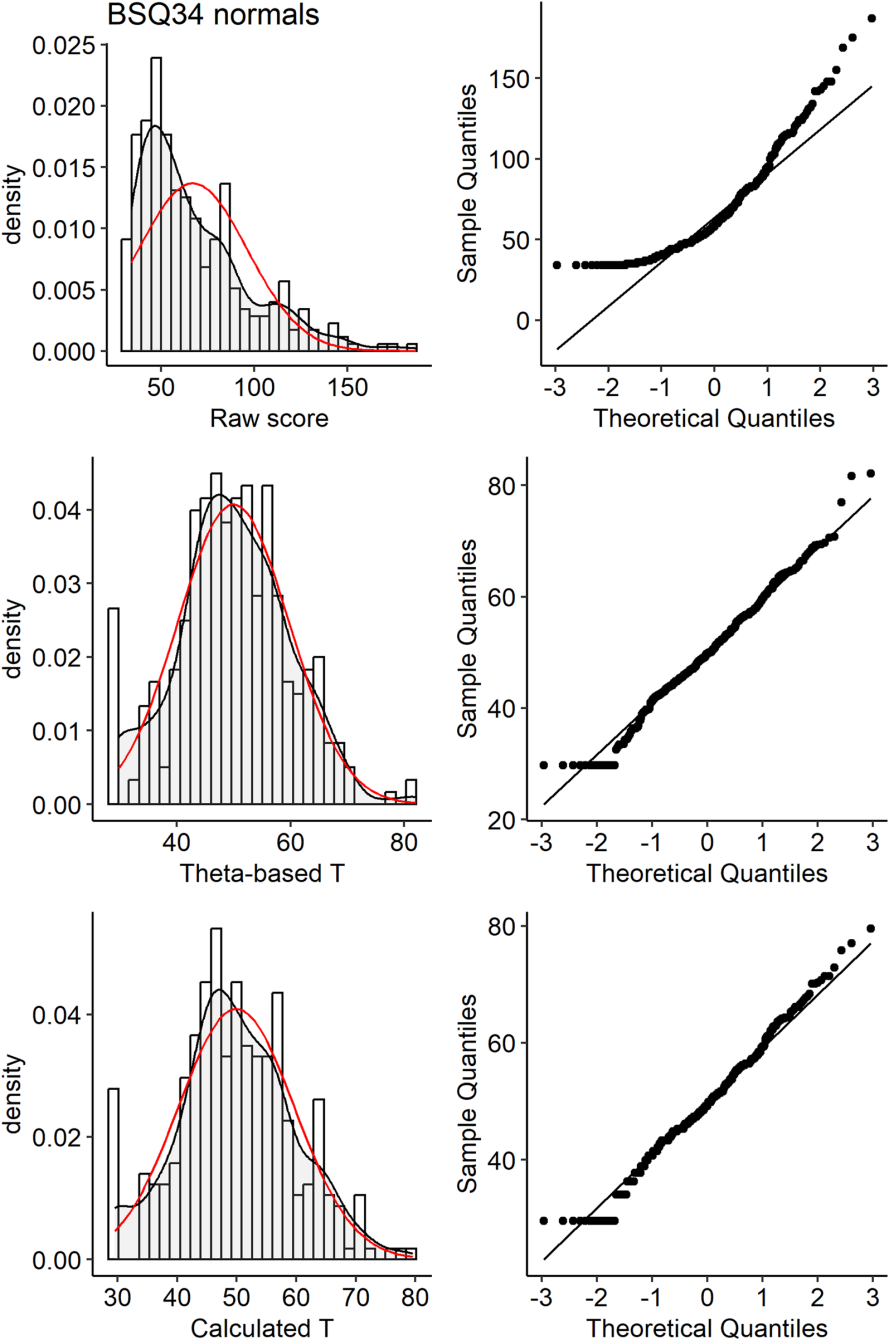
Histogram and normal probability plot for the BSQ34. Figure 2 shows that normalization was successful: the histograms reveals sufficient similarity between the density line (black) and a normal curve (red) and normal probability plots for raw scores and IRT-based T-scores for the BSQ34 also indicate normality, except for some overrepresentation of the lowest possible scores. IRT-based T-scores can be approximated by applying a curvilinear function to summed scale scores: For the BSQ34 this is *T* = 26.2 + 3.919*ln(RS-33.5) + 0.180*RS (RS = Raw scale score); Fig. 2 also shows successful normalization for T-scores based on this function. For the BSQ8C the function is *T* = −31.1 + 1.439e + 01*RS-1.029*RS^2 + 3.843e-02*RS^3–7.003e-04*RS^4 + 4.967e-06*RS^5

Figure 1 shows the relationship between the BSQ34 raw scores and theta-based *T*-scores. The figure shows some variance in *T*-scores per raw score (vertical dispersion), illustrating that summed scale scores corresponded to various factor scores. Figure 2 shows that normalization was successful by the histograms with sufficient similarity between the density line (black) and a normal curve (red) and normal probability plots for raw scores and *T*-scores of the BSQ34. IRT-based *T*-scores can be approximated by applying a curvilinear function to summed scale scores: For BSQ38 this is *T* = ax^3^ – bx^2^ (x = Raw scale score); For BSQ8C this was *T* = ax^3^ – bx^2^.

Percentile Rank (PR) scores were established based on the frequency of responses in the clinical sample, using: $PR=(\frac{m+0.5k}{N})*100$, where m is the number of respondents with a score < Raw Score (RS), k is the number of respondents with exactly RS and *N* is the size of the normative sample (Crawford & Garthwaite, 2009).

**Fig. 3:**
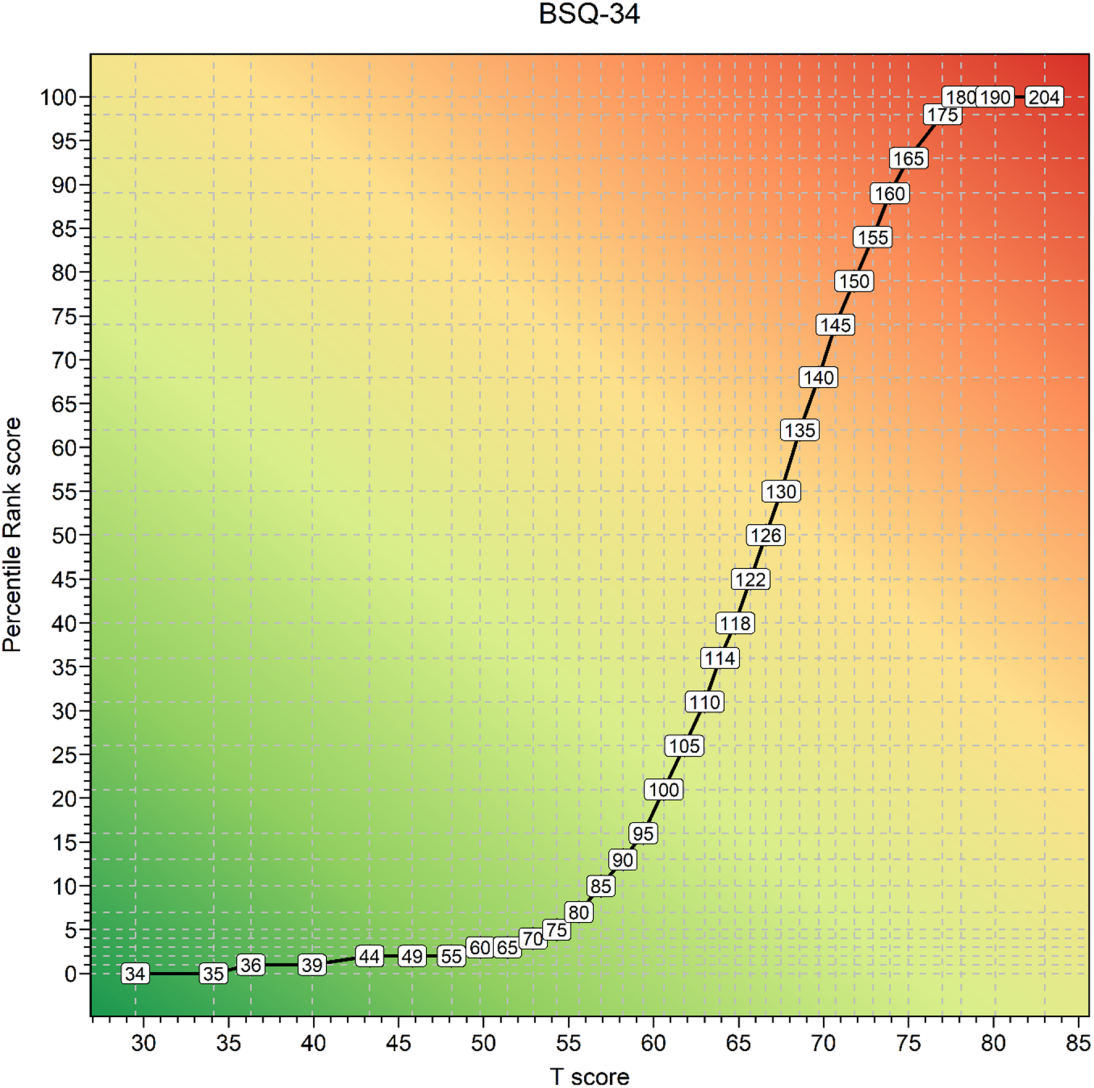
Raw scores, T-scores, and percentile rank scores of the Dutch BSQ34

For a selection of raw scores on the BSQ34 their association with *T*-scores and PR scores are presented in Fig. 3. In the supplementary materials cross-walk tables from all RS to *T*-scores and percentile rank scores are provided (Supplementary Tables B and C).

Finally, Table 3 shows that when the original cut-off score of > 110 [29] was applied to the BSQ34, a sensitivity of 70%, specificity 90% were found. However, the present data suggested different cut-off values for the Dutch culture. If optimal sensitivity was called for, e.g., when screening for subsequent assessment with a diagnostic interview, a raw score > 84.5 (*T* > 56.8) on the BSQ34 appears appropriate. When optimal specificity was called for, e.g., when screening for need of treatment, a cut-off of *RS* > 109.5 (*T* > 63) appears more appropriate. Sensitivity and specificity were balanced at 0.84 with a cutoff of *RS* > 97 (*T* > 59). Table 3 presents the BSQ8C cut-off values for the RS; corresponding cutoffs in *T*-scores are *T* > 49, *T* > 55 and *T* > 52.5. Figure 2**s**hows a cross-walk figure with percentile rank scores and *T*-scores for a selection of raw scores.

**Table 3:** Sensitivity and specificity for the Dutch Body Shape Questionnaire

		Optimal sensitivity			Optimal specificity			Best of both		
Scale	AUC	Cut-off	Sens	Spec	Cut-off	Sens	Spec	Cut-off	Sens	Spec
BSQ34	0.90	84.5	0.90	0.79	109.5	0.70	0.89	97	0.84	0.83
BSQ8C	0.91	13.5	0.90	0.80	18.5	0.73	0.90	16.5	0.88	0.86

*AUC* area under the curve, *Sens.* sensitivity, *Spec.* specificity

## Discussion

This is the first study to provide psychometric properties and norms of a Dutch version of the BSQ. The main findings from this study were that the Dutch version of the BSQ34 and the BSQ8C showed high internal consistency, concurrent validity, test–retest reliability, and incremental and criterion-related validity. In addition, both BSQs accurately discriminated in body-shape dissatisfaction between patients with BED and a community sample. Both BSQs showed sensitivity to change and had a unidimensional factor structure. However, the analysis of scalar invariance revealed that the items might be interpreted differently by patients with BED and respondents from the community sample. The cutoff of the BSQ34 was 97, representing a *T*-score of 59. Cut-off score for theBSQ8C was 16.5 corresponding with a *T*-score of 52.6. Consistent with previous research, the present study provides evidence that body-shape dissatisfaction is elevated among patients with BED [6].

Results regarding the psychometric properties of the Dutch BSQs were in accordance with several other studies [32–35, 59], which highlight the potential multi-cultural applicability of the BSQ34 and BSQ8C. It is suggested to use the BSQ34 as a first-line screening tool and subsequently determine if body-shape dissatisfaction should be addressed during BED treatment [5, 44]. Moreover, the BSQ8C could be used to measure treatment progression with regard to the reduction of body-shape dissatisfaction.

## Strengths and limits

This study has a variety of strengths. It is the first to investigate the properties of the Dutch BSQs in a sample of patients with BED and compare this to a large community sample. Various aspects of validity, reliability and the factor structure were examined. In addition, interview data were used to determine criterion-related validity, since interview data are more reliable than self-report data among eating disorder populations [60, 61]. Furthermore, this study contributes to the assessment and knowledge of body-shape dissatisfaction among patients with BED, which is very understudied relative to other eating disorder populations [62].

There are also certain limitations to this study. Firstly, BED remains underrecognized among a lot of patients, therefore, the sample might have been biased since only BED patients seeking treatment were included. Secondly, the community sample was also biased, as respondents of sample 2 were predominantly highly educated young women, and there were differences between the samples regarding gender, highest level of education, marital status and various eating disorder-related variables such as the scores on the self-report questionnaires. Therefore, further research examining the associations between BSQ scores and demographics is warranted. Consequently, various norm groups can be proposed. In addition, less than *n* = 100 men were included in the subsample of patients with BED. Therefore, a measurement invariance analysis for gender was not feasible [63, 64]. Furthermore, as found in the present study, women tend to report increased levels of body-shape dissatisfaction than men [65]. Consequently, the suggested cut-off scores should be used with some caution. The current estimated cut-off scores could be too high for men. In addition, unfortunately information on the severity of anxiety and depression was not available for the present samples. Consequently, anxiety and depression were prevented from being considered in the analyses. Furthermore, the sample might have been biased, since the participants in sample 2 likely had a special interest in (mental) health care, body-shape dissatisfaction or eating disorders. Therefore, they could have been more prone to report about their body-shape dissatisfaction or eating disorder pathology. Thirdly, the EDE-SC was used to determine how well the BSQs discriminated between the severity levels of body-shape dissatisfaction in an eating disorder sample. Use of the EDE subscale is not ideal since several studies have demonstrated that its factor structure is inconclusive [66–69], and its internal consistency in the current study was only acceptable for Cronbach’s *α*. The examination of the factor structure of the Dutch EDE [28] would be superior. However, only 155 patients with BED completed the EDE, therefore running a CFA in this sample would not yield valid results. The use of the EDE-SC appeared to be most suitable since bias appeared to be reduced due to its investigator-based nature [27]. Furthermore, among all subscales, the shape concern subscale had the highest internal consistency [69] and to date there are no other standardized measures available to measure body-shape dissatisfaction in the Netherlands. Fourth, data were collected in the context of a randomized controlled trial. Patients included in the trial were offered a version of the non-underweight treatment protocol of CBT-E, which is specifically designed for eating disorder patients with a BMI < 40 [44]. Therefore, only BED patients with a BMI < 40 were included, [70]. Thus, it is unknown how the BSQ performs with patients with BED with a BMI > 40. Fifth, the present study did not control for the effect of a high BMI among patients with BED. A high BMI could be associated with greater body-shape dissatisfaction and higher levels of eating disorder pathology [14, 71]. A comparison with a community sample without BED, but with a high BMI could estimate if the levels of body-shape dissatisfaction were related to the eating disorder, BMI, or both, and if body-shape dissatisfaction was higher among the patients with BED than in the community sample with a high BMI. Finally, based on the present study, the psychometric properties of the BSQ are yet unknown for other eating disorders such as anorexia and bulimia nervosa.

In the present study, one of the external criteria to evaluate the screening ability of the BSQs was a score above cut-off on the EDE-SC. Based on the current results, a logical next step for future research would be to examine the factor structure of the full Dutch EDE among a sufficient eating disorder sample. In addition, future studies are recommended to include measures of anxiety and depression severity to gain a more comprehensive understanding of their associations with body dissatisfaction. Furthermore, examination of the psychometric properties of the BSQs among patients with anorexia nervosa and bulimia nervosa is recommended. Future research should also consider examining the psychometric properties of additional measures that assess body-shape dissatisfaction, such as the Body Appreciation Scale-2 [72], Body Attitude Test [73], and the Body Uneasiness Test [74]. Both the Body Attitude Test, and Body Uneasiness Test have a stable multi-factor structure and examine various aspects of body-shape dissatisfaction. The Body Uneasiness Test, however, involves significantly more items compared to the BSQ34 and the Body Attitude Test is only moderately correlated with the BSQ34. In contrast to the other self-reports, the Body Appreciation Scale-2 measures a positive body image. Furthermore, it would also be of interest to investigate the psychometric properties of the Dutch BSQs among BED patients with a BMI > 40 and among a community sample with a high BMI. Finally, a more balanced community sample regarding gender, age, and educational level would increase confidence in the generalizability of the findings and normative values.

## Conclusion

In conclusion, this study evaluated the psychometric properties of the Dutch BSQs and provided normative data. For both the BSQ34 and the BSQ8C evidence of a unidimensional factor structure was found, and both have good psychometric properties, and can be considered suitable assessment tools to measure body-shape dissatisfaction among patients with BED. The estimated cut-off for a clinical severity level on the raw score for the BSQ34 was > 97 and > 16.5 for the BSQ8C. Community-based *T*-score equivalents are 59 and 52.6, respectively. However, respondents from the community may tend to interpret the items differently than patients with BED. In addition, young women were overrepresented in the community sample which potentially impacts the generalizability of the present findings regarding the Dutch BSQ negatively. Therefore, the results should be interpreted with care when the BSQs are applied in clinical practice.

## What is already known on this subject?

The BSQ assesses concerns about body-shape reported by patients with eating disorders. The BSQ has 34 items, but various short versions, measuring the same construct, have been developed and evaluated. The BSQ has a unidimensional factor structure, and strong psychometric properties, such as high test–retest reliability, internal validity, and convergent validity. The BSQ is adapted for use in various countries, but currently not available in Dutch for use in the Netherlands.

## What this study adds

The psychometric properties of the Dutch BSQ are satisfactory when administered among patients with BED. The ability to screen for body-shape dissatisfaction could help to assess if body-shape dissatisfaction is present in a BED patient. Subsequently, clinicians can decide whether body-shape dissatisfaction is an appropriate target for intervention within a broader BED treatment.

## Supplementary Information

Below is the link to the electronic supplementary material.

Supplementary material 1.

Supplementary material 2.

## Data Availability

Data are available upon reasonable request.
