# Supplementary material for: The Dutch Body Shape Questionnaire among patients with binge-eating disorder: psychometrics and norms of the full version (BSQ34) and the short version (BSQ8C)
Source: Eat Weight Disord. 2024 Nov 19;29(1):72. doi: 10.1007/s40519-024-01699-9 (PMC11576766; doi:10.1007/s40519-024-01699-9)
Supplement: Supplementary file 1 — Supplementary material 1. [file 40519_2024_1699_MOESM1_ESM.doc]

**BSQ-34**

Hoe heb je je de afgelopen VIER WEKEN gevoeld over je uiterlijk? Lees elke vraag en omcirkel rechts het corresponderende nummer. Beantwoord alstublieft alle vragen. GEDURENDE DE AFGELOPEN VIER WEKEN:

|  |  | Nooit | | | | | |  |
| --- | --- | --- | --- | --- | --- | --- | --- | --- |
|  |  | | | Zelden | | | | |  |
|  |  | | | | | Soms | | | |  |
|  |  | | | | | | | Vaak | | |  |
|  |  | | | | | | | | | Heel vaak | | |
|  |  | | | | | | | | | | | Altijd | |
|  |  | | | | | | | | | | | | |  |
| 1. | Heb je uit verveling gepiekerd over je figuur?......................................... | 1 | 2 | 3 | 4 | 5 | 6 |  |
| 2. | Heb je je zoveel zorgen gemaakt over je figuur dat je het gevoel had dat je op dieet moest?............................................................................... | 1 | 2 | 3 | 4 | 5 | 6 |  |
| 3. | Vond je dat je dijen, heupen of billen in verhouding met de rest van je lichaam te groot zijn? .............................................................................. | 1 | 2 | 3 | 4 | 5 | 6 |  |
| 4. | Ben je bang geweest dat je dik (of dikker) zou worden? ........................ | 1 | 2 | 3 | 4 | 5 | 6 |  |
| 5. | Maakte je je zorgen dat bepaalde delen van je lichaam niet strak genoeg zijn? | 1 | 2 | 3 | 4 | 5 | 6 |  |
| 6. | Heb je jezelf dik gevoeld door een vol gevoel (bijv. na het eten van een grote maaltijd)?......................................................................................... | 1 | 2 | 3 | 4 | 5 | 6 |  |
| 7. | Heb je jezelf zo slecht gevoeld over je figuur dat je erdoor moest huilen?...................................................................................................... | 1 | 2 | 3 | 4 | 5 | 6 |  |
| 8. | Heb je rennen vermeden omdat bepaalde delen van je lichaam zouden kunnen schudden? | 1 | 2 | 3 | 4 | 5 | 6 |  |
| 9. | Heeft het samenzijn met dunne mensen ervoor gezorgd dat je jezelf meer bewust werd van je figuur? ............................................................. | 1 | 2 | 3 | 4 | 5 | 6 |  |
| 10. | Maakte je je zorgen over dat je bovenbenen zich verbreden wanneer je gaat zitten? ............................................................................................... | 1 | 2 | 3 | 4 | 5 | 6 |  |
| 11. | Heeft het eten van zelfs maar een kleine hoeveelheid voedsel je een dik gevoel gegeven? ....................................................................................... | 1 | 2 | 3 | 4 | 5 | 6 |  |
| 12. | Heb je bij het zien van het figuur van anderen geconcludeerd dat je een minder mooi figuur hebt in vergelijking tot hen?..................................... | 1 | 2 | 3 | 4 | 5 | 6 |  |
| 13. | Heeft het nadenken over je figuur je concentratievermogen belemmerd (bijv. terwijl je televisie kijkt, leest, naar gesprekken luistert)?............... | 1 | 2 | 3 | 4 | 5 | 6 |  |
| 14. | Heb je je weleens dik gevoeld wanneer je naakt was, bijvoorbeeld in bad of onder de douche? .......................................................................... | 1 | 2 | 3 | 4 | 5 | 6 |  |
| 15. | Heb je het vermeden kleding te dragen die je nog bewuster maakt van je figuur? .................................................................................................. | 1 | 2 | 3 | 4 | 5 | 6 |  |
| 16. | Heb je je weleens ingebeeld dat je stukken huid of vet van je lichaam af sneed?................................................................................................... | 1 | 2 | 3 | 4 | 5 | 6 |  |

|  |  | Nooit | | | | | |  | |
| --- | --- | --- | --- | --- | --- | --- | --- | --- | --- |
|  |  | | | Zelden | | | | |  | |
|  |  | | | | | Soms | | | |  | |
|  |  | | | | | | | Vaak | | |  | |
|  |  | | | | | | | | | Heel vaak | | |  |
|  |  | | | | | | | | | | | Altijd | | |
|  |  | | | | | | | | | | | | |  | |
| 17. | Heb je je dik gevoeld door het eten van snoep, taart of ander hoog calorisch voedsel? .................................................................................... | 1 | 2 | 3 | 4 | 5 | 6 |  | |
| 18. | Ben je niet naar sociale gelegenheden (bijv. feestjes) gegaan omdat je je slecht voelde over je figuur? ................................................................ | 1 | 2 | 3 | 4 | 5 | 6 |  | |
| 19. | Voelde je je buitenproportioneel groot en rond?...................................... | 1 | 2 | 3 | 4 | 5 | 6 |  | |
| 20. | Heb je je geschaamd voor je lichaam? .................................................... | 1 | 2 | 3 | 4 | 5 | 6 |  | |
| 21. | Hebben de zorgen die je je gemaakt hebt over je figuur, je aangezet tot dieten?...................................................................................................... | 1 | 2 | 3 | 4 | 5 | 6 |  | |
| 22. | Was je met een lege maag (bijv. ’s morgens) het meest gelukkig met je figuur? ...................................................................................................... | 1 | 2 | 3 | 4 | 5 | 6 |  | |
| 23. | Heb je gedacht dat je dit figuur hebt, wegens een gebrek aan zelfbeheersing? ........................................................................................ | 1 | 2 | 3 | 4 | 5 | 6 |  | |
| 24. | Heb je je zorgen gemaakt dat andere mensen vetrollen rond je middel of buik zien? ............................................................................................ | 1 | 2 | 3 | 4 | 5 | 6 |  | |
| 25. | Heb je het gevoel gehad dat het niet eerlijk is dat andere mensen dunner zijn dan jij? .................................................................................. | 1 | 2 | 3 | 4 | 5 | 6 |  | |
| 26. | Heb je overgegeven om je dunner te voelen? .......................................... | 1 | 2 | 3 | 4 | 5 | 6 |  | |
| 27. | Heb je in gezelschap van anderen je zorgen gemaakt dat je te veel ruimte in zou nemen (bijvoorbeeld toen je op de bank zat of in de bus)? | 1 | 2 | 3 | 4 | 5 | 6 |  | |
| 28. | Piekerde je over de putjes in je huid? ...................................................... | 1 | 2 | 3 | 4 | 5 | 6 |  | |
| 29. | Heeft het zien van je spiegelbeeld (bijvoorbeeld in een spiegel of etalage) je een slecht gevoel gegeven over je figuur?.............................. | 1 | 2 | 3 | 4 | 5 | 6 |  | |
| 30. | Heb je in delen van je lichaam geknepen om te zien hoeveel vet er is? | 1 | 2 | 3 | 4 | 5 | 6 |  | |
| 31. | Heb je situaties vermeden waarin mensen je lichaam konden zien (bijv. gemeenschappelijke kleedkamers of het zwembad)?............................... | 1 | 2 | 3 | 4 | 5 | 6 |  | |
| 32. | Heb je laxeermiddelen gebruikt om je dunner te voelen?........................ | 1 | 2 | 3 | 4 | 5 | 6 |  | |
| 33. | Ben je in het bijzijn van anderen meer bijzonder bewust geweest over je figuur dan anders? …………………………………………………… | 1 | 2 | 3 | 4 | 5 | 6 |  | |
| 34. | Vond je dat je moest gaan bewegen wegens zorgen om je figuur?........... | 1 | 2 | 3 | 4 | 5 | 6 |  | |
