# Supplementary material for: The Dutch Body Shape Questionnaire among patients with binge-eating disorder: psychometrics and norms of the full version (BSQ34) and the short version (BSQ8C)
Source: Eat Weight Disord. 2024 Nov 19;29(1):72. doi: 10.1007/s40519-024-01699-9 (PMC11576766; doi:10.1007/s40519-024-01699-9)
Supplement: Supplementary file 2 — Supplementary material 2. [file 40519_2024_1699_MOESM2_ESM.docx]

**Supplementary Table A1**

Basic psychometrics of the BSQ items and total scores in the general population

|  |  |  |  |  |  |  |  |  |  |  |  |  |
| --- | --- | --- | --- | --- | --- | --- | --- | --- | --- | --- | --- | --- |
| Items | *n* | *M* | SD | median | skewness | kurtosis | 1 | 2 | 3 | 4 | 5 | 6 |
|  |  |  |  |  |  |  |  |  |  |  |  |  |
| Item1 | 333 | 2.14 | 1.17 | 2 | 0.90 | 0.30 | 38 | 28 | 21 | 10 | 02 | 01 |
| Item2 | 333 | 2.36 | 1.44 | 2 | 0.91 | -0.03 | 38 | 21 | 20 | 10 | 05 | 05 |
| Item3 | 333 | 1.99 | 1.35 | 1 | 1.31 | 0.89 | 54 | 16 | 17 | 06 | 05 | 03 |
| Item4 | 333 | 2.74 | 1.34 | 3 | 0.59 | -0.12 | 21 | 23 | 33 | 12 | 06 | 05 |
| Item5 | 333 | 2.89 | 1.38 | 3 | 0.44 | -0.48 | 18 | 23 | 29 | 16 | 08 | 05 |
| Item6 | 333 | 2.76 | 1.40 | 3 | 0.48 | -0.46 | 24 | 19 | 32 | 13 | 08 | 05 |
| Item7 | 333 | 1.42 | 0.94 | 1 | 2.63 | 6.96 | 77 | 12 | 06 | 02 | 02 | 01 |
| Item8 | 333 | 1.24 | 0.78 | 1 | **3.71** | **13.94** | 89 | 05 | 03 | 02 | 02 | 00 |
| Item9 | 333 | 2.11 | 1.43 | 1 | 1.17 | 0.42 | 51 | 16 | 17 | 08 | 05 | 04 |
| Item10 | 333 | 1.82 | 1.28 | 1 | 1.5 | 1.29 | 62 | 14 | 11 | 06 | 05 | 02 |
| Item11 | 333 | 1.56 | 0.98 | 1 | **1.94** | **3.93** | 69 | 14 | 14 | 02 | 01 | 01 |
| Item12 | 333 | 2.69 | 1.33 | 3 | 0.48 | -0.44 | 23 | 22 | 31 | 12 | 09 | 03 |
| Item13 | 333 | 1.39 | 0.85 | 1 | 2.45 | 6.26 | 78 | 10 | 09 | 02 | 01 | 00 |
| Item14 | 333 | 2.52 | 1.35 | 2 | 0.66 | -0.18 | 30 | 21 | 30 | 10 | 06 | 03 |
| Item15 | 333 | 2.44 | 1.35 | 2 | 0.67 | -0.28 | 34 | 20 | 26 | 13 | 05 | 03 |
| Item16 | 333 | 1.36 | 0.88 | 1 | **2.77** | **7.91** | 81 | 08 | 07 | 02 | 01 | 01 |
| Item17 | 333 | 2.38 | 1.40 | 2 | 0.73 | -0.32 | 38 | 17 | 23 | 14 | 05 | 03 |
| Item18 | 333 | 1.16 | 0.55 | 1 | **4.45** | **24.64** | 89 | 07 | 03 | 01 | 00 | 00 |
| Item19 | 333 | 1.56 | 1.04 | 1 | **2.12** | **4.42** | 70 | 14 | 10 | 03 | 02 | 01 |
| Item20 | 333 | 2.12 | 1.21 | 2 | 0.93 | 0.38 | 43 | 20 | 26 | 07 | 03 | 02 |
| Item21 | 333 | 2.04 | 1.31 | 1 | **1.15** | 0.54 | 50 | 18 | 17 | 09 | 03 | 02 |
| Item22 | 333 | 2.19 | 1.52 | 1 | **1.07** | 0.01 | 52 | 14 | 14 | 11 | 05 | 05 |
| Item23 | 333 | 2.08 | 1.43 | 1 | **1.18** | 0.43 | 53 | 14 | 17 | 08 | 04 | 04 |
| Item24 | 333 | 2.34 | 1.35 | 2 | 0.82 | -0.09 | 36 | 23 | 23 | 10 | 06 | 03 |
| Item25 | 333 | 1.64 | 1.05 | 1 | **1.79** | 3.17 | 66 | 14 | 15 | 03 | 01 | 01 |
| Item26 | 333 | 1.13 | 0.55 | 1 | **4.91** | **25.02** | 94 | 03 | 02 | 02 | 01 | 00 |
| Item27 | 333 | 1.31 | 0.83 | 1 | **3.15** | **10.45** | 84 | 07 | 05 | 02 | 01 | 01 |
| Item28 | 333 | 1.62 | 1.06 | 1 | **1.94** | **3.64** | 66 | 16 | 12 | 03 | 02 | 01 |
| Item29 | 333 | 2.20 | 1.24 | 2 | 0.95 | 0.36 | 38 | 26 | 23 | 08 | 04 | 02 |
| Item30 | 333 | 2.40 | 1.26 | 2 | 0.56 | -0.43 | 32 | 21 | 29 | 11 | 05 | 01 |
| Item31 | 333 | 1.57 | 1.04 | 1 | **2.15** | 4.75 | 69 | 15 | 10 | 03 | 01 | 02 |
| Item32 | 333 | 1.05 | 0.36 | 1 | **8.07** | **69.35** | 98 | 01 | 01 | 01 | 00 | 00 |
| Item33 | 333 | 2.15 | 1.22 | 2 | 0.79 | -0.22 | 42 | 21 | 21 | 11 | 03 | 01 |
| Item34 | 333 | 2.68 | 1.41 | 3 | 0.56 | -0.41 | 26 | 21 | 28 | 14 | 06 | 05 |
| BSQ34 | 333 | 67.06 | 29.17 | 58 | **1.24** | **1.37** |  |  |  |  |  |  |
| BSQ8C | 333 | 16.24 | 7.50 | 14 | **1.24** | **1.31** |  |  |  |  |  |  |

† The indicators marked in bold type signify skewed and peaked items with many responses in the lowest response category.

**Supplementary Table A2**

Basic psychometrics of the BSQ items and total scores in the clinical sample

|  |  |  |  |  |  |  |  |  |  |  |  |  |
| --- | --- | --- | --- | --- | --- | --- | --- | --- | --- | --- | --- | --- |
| Items | *n* | *M* | SD | median | skewness | kurtosis | 1 | 2 | 3 | 4 | 5 | 6 |
|  |  |  |  |  |  |  |  |  |  |  |  |  |
| Item1 | 155 | 3.83 | 1.45 | 4 | -0.28 | -0.75 | 08 | 10 | 22 | 25 | 22 | 14 |
| Item2 | 155 | 4.81 | 1.14 | 5 | -0.79 | -0.04 | 01 | 03 | 12 | 17 | 34 | 33 |
| Item3 | 155 | 3.99 | 1.83 | 4 | -0.38 | -1.29 | 15 | 12 | 10 | 17 | 15 | 31 |
| Item4 | 155 | 5.02 | 1.16 | 5 | -0.94 | -0.23 | 00 | 03 | 11 | 14 | 24 | 48 |
| Item5 | 155 | 4.66 | 1.38 | 5 | -0.84 | -0.14 | 03 | 05 | 12 | 20 | 23 | 37 |
| Item6 | 155 | 4.46 | 1.22 | 5 | -0.50 | -0.49 | 01 | 05 | 18 | 22 | 32 | 23 |
| Item7 | 155 | 2.95 | 1.43 | 3 | 0.25 | -0.82 | 21 | 15 | 33 | 13 | 14 | 04 |
| Item8 | 155 | 2.90 | 1.73 | 3 | 0.44 | -1.13 | 31 | 16 | 19 | 11 | 12 | 11 |
| Item9 | 155 | 4.33 | 1.46 | 4 | -0.57 | -0.52 | 06 | 05 | 18 | 23 | 21 | 28 |
| Item10 | 155 | 3.35 | 1.71 | 3 | 0.06 | -1.23 | 21 | 12 | 21 | 16 | 15 | 14 |
| Item11 | 155 | 2.61 | 1.3 | 2 | 0.77 | 0.11 | 20 | 33 | 26 | 11 | 06 | 04 |
| Item12 | 155 | 4.54 | 1.27 | 5 | -0.73 | -0.08 | 03 | 03 | 17 | 18 | 34 | 25 |
| Item13 | 155 | 3.01 | 1.41 | 3 | 0.28 | -0.82 | 16 | 24 | 24 | 19 | 12 | 05 |
| Item14 | 155 | 4.90 | 1.17 | 5 | -0.81 | -0.19 | 01 | 02 | 12 | 18 | 26 | 41 |
| Item15 | 155 | 4.77 | 1.14 | 5 | -0.62 | -0.24 | 01 | 03 | 10 | 28 | 25 | 34 |
| Item16 | 155 | 2.19 | 1.55 | 1 | 1.04 | -0.18 | 53 | 12 | 14 | 08 | 08 | 05 |
| Item17 | 155 | 4.46 | 1.20 | 5 | -0.56 | -0.14 | 02 | 03 | 16 | 26 | 31 | 22 |
| Item18 | 155 | 2.52 | 1.47 | 2 | 0.73 | -0.5 | 32 | 26 | 19 | 10 | 10 | 04 |
| Item19 | 155 | 4.08 | 1.43 | 4 | -0.33 | -0.74 | 05 | 09 | 20 | 25 | 21 | 20 |
| Item20 | 155 | 4.54 | 1.27 | 5 | -0.84 | 0.15 | 03 | 03 | 17 | 15 | 38 | 24 |
| Item21 | 155 | 3.66 | 1.61 | 4 | -0.24 | -1.17 | 14 | 13 | 19 | 14 | 28 | 12 |
| Item22 | 155 | 2.96 | 1.69 | 3 | 0.33 | -1.18 | 29 | 16 | 16 | 17 | 12 | 10 |
| Item23 | 155 | 4.83 | 1.26 | 5 | **-1.22** | 1.2 | 04 | 01 | 10 | 15 | 33 | 37 |
| Item24 | 155 | 4.63 | 1.37 | 5 | **-1.03** | 0.5 | 05 | 03 | 09 | 21 | 30 | 32 |
| Item25 | 155 | 3.26 | 1.63 | 3 | 0.23 | -1.08 | 17 | 18 | 24 | 15 | 13 | 13 |
| Item26 | 155 | 1.09 | 0.47 | 1 | **7.86** | **72.98** | 94 | 05 | 01 | 00 | 00 | 01 |
| Item27 | 155 | 3.15 | 1.64 | 3 | 0.17 | -1.12 | 24 | 12 | 24 | 17 | 14 | 10 |
| Item28 | 155 | 2.68 | 1.42 | 3 | 0.5 | -0.62 | 27 | 21 | 25 | 15 | 08 | 04 |
| Item29 | 155 | 4.42 | 1.24 | 5 | -0.46 | -0.4 | 02 | 04 | 18 | 26 | 27 | 23 |
| Item30 | 155 | 3.28 | 1.47 | 3 | -0.03 | -1.01 | 17 | 10 | 33 | 12 | 23 | 05 |
| Item31 | 155 | 3.14 | 1.63 | 3 | 0.28 | -1.1 | 20 | 19 | 23 | 13 | 15 | 10 |
| Item32 | 155 | 1.13 | 0.54 | 1 | **4.92** | **25.81** | 93 | 04 | 01 | 01 | 01 | 00 |
| Item33 | 155 | 4.00 | 1.30 | 4 | -0.32 | -0.46 | 04 | 10 | 18 | 33 | 22 | 14 |
| Item34 | 155 | 4.07 | 1.35 | 4 | -0.46 | -0.34 | 06 | 06 | 19 | 29 | 25 | 15 |
| BSQ34 | 155 | 124.25 | 28.54 | 124 | -0.27 | -0.56 |  |  |  |  |  |  |
| BSQ8C | 155 | 32.01 | 6.90 | 32 | -0.24 | -0.52 |  |  |  |  |  |  |

† The indicators marked in bold type signify skewed and peaked items with many responses in the lowest response category.

**Supplementary Table B**

Cross walk from raw scores to *T*-scores and percentiles for the Dutch BSQ34

| RS^1^ | T^2^ | P_n^3^ | P_cl^4^ |  | RS^1^ | T^2^ | P_n^3^ | P_cl^4^ |  | RS^1^ | T^2^ | P_n^3^ | P_cl^4^ |  | RS^1^ | T^2^ | P_n^3^ | P_cl^4^ |
| --- | --- | --- | --- | --- | --- | --- | --- | --- | --- | --- | --- | --- | --- | --- | --- | --- | --- | --- |
| 34 | 29.5 | 2 | 0 |  | 77 | 54.8 | 71 | 6 |  | 120 | 65.2 | 93 | 43 |  | 163 | 74.5 | 99 | 91 |
| 35 | 34.1 | 4 | 0 |  | 78 | 55.1 | 72 | 6 |  | 121 | 65.5 | 94 | 44 |  | 164 | 74.8 | 99 | 92 |
| 36 | 36.3 | 7 | 1 |  | 79 | 55.4 | 73 | 7 |  | 122 | 65.7 | 94 | 45 |  | 165 | 75.0 | 99 | 93 |
| 37 | 37.8 | 9 | 1 |  | 80 | 55.6 | 73 | 7 |  | 123 | 65.9 | 94 | 46 |  | 166 | 75.2 | 100 | 94 |
| 38 | 38.9 | 11 | 1 |  | 81 | 55.9 | 74 | 8 |  | 124 | 66.1 | 94 | 48 |  | 167 | 75.4 | 100 | 94 |
| 39 | 39.9 | 13 | 1 |  | 82 | 56.2 | 75 | 8 |  | 125 | 66.4 | 94 | 49 |  | 168 | 75.6 | 100 | 95 |
| 40 | 40.7 | 16 | 2 |  | 83 | 56.4 | 76 | 8 |  | 126 | 66.6 | 95 | 50 |  | 169 | 75.8 | 100 | 96 |
| 41 | 41.5 | 18 | 2 |  | 84 | 56.7 | 77 | 9 |  | 127 | 66.8 | 95 | 52 |  | 170 | 76.0 | 100 | 96 |
| 42 | 42.1 | 20 | 2 |  | 85 | 56.9 | 77 | 10 |  | 128 | 67.0 | 95 | 53 |  | 171 | 76.2 | 100 | 97 |
| 43 | 42.8 | 22 | 2 |  | 86 | 57.2 | 78 | 10 |  | 129 | 67.2 | 95 | 54 |  | 172 | 76.4 | 100 | 97 |
| 44 | 43.3 | 24 | 2 |  | 87 | 57.4 | 79 | 11 |  | 130 | 67.5 | 96 | 55 |  | 173 | 76.6 | 100 | 98 |
| 45 | 43.9 | 26 | 2 |  | 88 | 57.7 | 79 | 11 |  | 131 | 67.7 | 96 | 57 |  | 174 | 76.8 | 100 | 98 |
| 46 | 44.4 | 28 | 2 |  | 89 | 57.9 | 80 | 12 |  | 132 | 67.9 | 96 | 58 |  | 175 | 77 | 100 | 98 |
| 47 | 44.9 | 30 | 2 |  | 90 | 58.2 | 81 | 13 |  | 133 | 68.1 | 96 | 59 |  | 176 | 77.3 | 100 | 99 |
| 48 | 45.3 | 32 | 2 |  | 91 | 58.4 | 81 | 13 |  | 134 | 68.3 | 96 | 60 |  | 177 | 77.5 | 100 | 99 |
| 49 | 45.8 | 34 | 2 |  | 92 | 58.7 | 82 | 14 |  | 135 | 68.6 | 96 | 62 |  | 178 | 77.7 | 100 | 99 |
| 50 | 46.2 | 35 | 2 |  | 93 | 58.9 | 83 | 15 |  | 136 | 68.8 | 96 | 63 |  | 179 | 77.9 | 100 | 100 |
| 51 | 46.6 | 37 | 2 |  | 94 | 59.2 | 83 | 16 |  | 137 | 69.0 | 97 | 64 |  | 180 | 78.1 | 100 | 100 |
| 52 | 47.0 | 39 | 2 |  | 95 | 59.4 | 84 | 16 |  | 138 | 69.2 | 97 | 65 |  | 181 | 78.3 | 100 | 100 |
| 53 | 47.4 | 40 | 2 |  | 96 | 59.7 | 84 | 17 |  | 139 | 69.4 | 97 | 67 |  | 182 | 78.5 | 100 | 100 |
| 54 | 47.7 | 42 | 2 |  | 97 | 59.9 | 85 | 18 |  | 140 | 69.7 | 97 | 68 |  | 183 | 78.7 | 100 | 100 |
| 55 | 48.1 | 44 | 2 |  | 98 | 60.1 | 85 | 19 |  | 141 | 69.9 | 97 | 69 |  | 184 | 78.9 | 100 | 100 |
| 56 | 48.5 | 45 | 2 |  | 99 | 60.4 | 86 | 20 |  | 142 | 70.1 | 97 | 70 |  | 185 | 79.1 | 100 | 100 |
| 57 | 48.8 | 47 | 2 |  | 100 | 60.6 | 86 | 21 |  | 143 | 70.3 | 97 | 72 |  | 186 | 79.3 | 100 | 100 |
| 58 | 49.2 | 48 | 2 |  | 101 | 60.9 | 87 | 22 |  | 144 | 70.5 | 98 | 73 |  | 187 | 79.5 | 100 | 100 |
| 59 | 49.5 | 50 | 3 |  | 102 | 61.1 | 87 | 23 |  | 145 | 70.7 | 98 | 74 |  | 188 | 79.7 | 100 | 100 |
| 60 | 49.8 | 51 | 3 |  | 103 | 61.3 | 88 | 24 |  | 146 | 70.9 | 98 | 75 |  | 189 | 79.9 | 100 | 100 |
| 61 | 50.2 | 53 | 3 |  | 104 | 61.6 | 88 | 25 |  | 147 | 71.2 | 98 | 76 |  | 190 | 80.1 | 100 | 100 |
| 62 | 50.5 | 54 | 3 |  | 105 | 61.8 | 88 | 26 |  | 148 | 71.4 | 98 | 77 |  | 191 | 80.3 | 100 | 100 |
| 63 | 50.8 | 55 | 3 |  | 106 | 62.0 | 89 | 27 |  | 149 | 71.6 | 98 | 78 |  | 192 | 80.5 | 100 | 100 |
| 64 | 51.1 | 57 | 3 |  | 107 | 62.3 | 89 | 28 |  | 150 | 71.8 | 98 | 79 |  | 193 | 80.8 | 100 | 100 |
| 65 | 51.4 | 58 | 3 |  | 108 | 62.5 | 90 | 29 |  | 151 | 72.0 | 98 | 80 |  | 194 | 81.0 | 100 | 100 |
| 66 | 51.7 | 59 | 3 |  | 109 | 62.7 | 90 | 30 |  | 152 | 72.2 | 98 | 82 |  | 195 | 81.2 | 100 | 100 |
| 67 | 52.0 | 60 | 3 |  | 110 | 63.0 | 90 | 31 |  | 153 | 72.4 | 98 | 82 |  | 196 | 81.4 | 100 | 100 |
| 68 | 52.3 | 62 | 4 |  | 111 | 63.2 | 91 | 32 |  | 154 | 72.6 | 99 | 84 |  | 197 | 81.6 | 100 | 100 |
| 69 | 52.6 | 63 | 4 |  | 112 | 63.4 | 91 | 33 |  | 155 | 72.9 | 99 | 84 |  | 198 | 81.8 | 100 | 100 |
| 70 | 52.9 | 64 | 4 |  | 113 | 63.7 | 91 | 34 |  | 156 | 73.1 | 99 | 86 |  | 199 | 82.0 | 100 | 100 |
| 71 | 53.2 | 65 | 4 |  | 114 | 63.9 | 92 | 36 |  | 157 | 73.3 | 99 | 86 |  | 200 | 82.2 | 100 | 100 |
| 72 | 53.5 | 66 | 4 |  | 115 | 64.1 | 92 | 37 |  | 158 | 73.5 | 99 | 87 |  | 201 | 82.4 | 100 | 100 |
| 73 | 53.7 | 67 | 5 |  | 116 | 64.3 | 92 | 38 |  | 159 | 73.7 | 99 | 88 |  | 202 | 82.6 | 100 | 100 |
| 74 | 54.0 | 68 | 5 |  | 117 | 64.6 | 92 | 39 |  | 160 | 73.9 | 99 | 89 |  | 203 | 82.8 | 100 | 100 |
| 75 | 54.3 | 69 | 5 |  | 118 | 64.8 | 93 | 40 |  | 161 | 74.1 | 99 | 90 |  | 204 | 83.0 | 100 | 100 |
| 76 | 54.6 | 70 | 6 |  | 119 | 65.0 | 93 | 42 |  | 162 | 74.3 | 99 | 91 |  |  |  |  |  |

^1^ Raw scores;

^2^ Calculated *T*-scores based on IRT (*T* =26.2+3.919*ln(RS-33.5)+0.180*RS, a logarithmic function + a linear term);

^3^ Calculated Percentile Rank Scores for the general population (*T*=-283.2+384.5/(1+EXP(-3.252e-02*(RS-1.6))); a sigmoid function).

^4^ Calculated Percentile Rank Scores for the clinical sample (*T*=-62.5+4.135*RS-9.728e-02*RS^2+1.010e-03*RS^3-4.307e-06*RS^4+6.371e-09*RS^5; a polynomial function).

**Supplementary Table C**

Cross walk from raw scores to *T*-scores and Percentile ranks scores

for the Dutch BSQ8C.

| RS^1^ | T^2^ | P_n^3^ | P_cl^4^ |  | RS^1^ | T^2^ | P_n^3^ | P_cl^4^ |  | RS^1^ | T^2^ | P_n^3^ | P_cl^4^ |
| --- | --- | --- | --- | --- | --- | --- | --- | --- | --- | --- | --- | --- | --- |
| 8 | 35.0 | 3 | 0 |  | 22 | 57.8 | 81 | 8 |  | 36 | 69.6 | 97 | 69 |
| 9 | 38.7 | 12 | 1 |  | 23 | 58.7 | 83 | 11 |  | 37 | 70.3 | 98 | 74 |
| 10 | 41.7 | 21 | 1 |  | 24 | 59.6 | 85 | 14 |  | 38 | 71.0 | 98 | 78 |
| 11 | 44.3 | 29 | 1 |  | 25 | 60.5 | 87 | 17 |  | 39 | 71.7 | 98 | 83 |
| 12 | 46.4 | 36 | 1 |  | 26 | 61.4 | 89 | 21 |  | 40 | 72.5 | 99 | 87 |
| 13 | 48.2 | 43 | 1 |  | 27 | 62.3 | 90 | 25 |  | 41 | 73.3 | 99 | 90 |
| 14 | 49.7 | 49 | 1 |  | 28 | 63.2 | 91 | 29 |  | 42 | 74.2 | 99 | 93 |
| 15 | 51.1 | 54 | 1 |  | 29 | 64.1 | 92 | 34 |  | 43 | 75.4 | 100 | 96 |
| 16 | 52.2 | 59 | 1 |  | 30 | 65.0 | 94 | 38 |  | 44 | 76.7 | 100 | 98 |
| 17 | 53.3 | 64 | 2 |  | 31 | 65.8 | 94 | 43 |  | 45 | 78.3 | 100 | 99 |
| 18 | 54.3 | 68 | 2 |  | 32 | 66.6 | 95 | 48 |  | 46 | 80.2 | 100 | 100 |
| 19 | 55.2 | 72 | 3 |  | 33 | 67.4 | 96 | 53 |  | 47 | 82.6 | 100 | 100 |
| 20 | 56.1 | 75 | 5 |  | 34 | 68.2 | 96 | 59 |  | 48 | 85.6 | 100 | 100 |
| 21 | 57.0 | 78 | 6 |  | 35 | 68.9 | 97 | 64 |  |  |  |  |  |

1 Raw scores;

2 Calculated T-scores based on IRT (*T*=-31.1+1.439e+01*RS-1.029*RS^2+3.843e-02*RS^3-7.003e-04*RS^4+4.967e-06*RS^5; a poly5 function);

3 Calculated Percentile Rank Scores for the general population (*T*=-270.2+371.0/(1+EXP(-1.305e-01*(RS-0.1))); a polynominal function);

4 Calculated Percentile Rank Scores for the clinical sample (*T*=-45.4+1.246e+01*RS-1.191*RS^2+4.850e-02*RS^3-7.718e-04*RS^4+4.019e-06*RS^5; a polynominal function).
